# Desphospho-Uncarboxylated Matrix-Gla Protein Is Increased Postoperatively in Cardiovascular Risk Patients

**DOI:** 10.3390/nu10010046

**Published:** 2018-01-05

**Authors:** Sofia Dahlberg, Jacob Ede, Leon Schurgers, Cees Vermeer, Thomas Kander, Bengt Klarin, Ulf Schött

**Affiliations:** 1 Institution of Clinical Science Lund, Medical Faculty, Lund University, S-22185 Lund, Sweden; sofia.irma.dahlberg@gmail.com (S.D.); jacob.ede@skane.se (J.E.); thomas.kander@skane.se (T.K.); bengt.klarin@skane.se (B.K.); 2 Department of Biochemistry, CARIM School for Cardiovascular Diseases, Maastricht University, Universiteitssingel 50, 6229 ER Maastricht, The Netherlands; l.schurgers@maastrichtuniversity.nl; 3 R&D Group VitaK, Maastricht University, Oxfordlaan 55, 6229 ER Maastricht, The Netherlands; cees.vermeer@outlook.com; 4 Department of Anaesthesiology and Intensive Care, Institution of Clinical Sciences Lund, Lund University and Skane University Hospital, S-22185 Lund, Sweden

**Keywords:** atherosclerosis, malnutrition, matrix Gla protein, osteocalcin, surgery, vitamin K

## Abstract

Background: Matrix Gla protein (MGP) is an extrahepatic protein that is dependent on glutamate carboxylation, a vitamin K-dependent process. Its dysfunctional form, desphospho-uncarboxylated-MGP, has been associated with increased arterial calcification and stiffness. The aim of this study was to measure the degree of postoperative carboxylation of MGP and two other Gla proteins in patients scheduled for abdominal or orthopaedic surgery. Methods: Forty patients undergoing abdominal or orthopaedic surgery were included. Blood samples were collected preoperatively and four days after the surgery. Desphospho-carboxylated MGP (dp-cMGP), desphospho-uncarboxylated MGP (dp-ucMGP), carboxylated osteocalcin (OC) (cOC), uncarboxylated OC (ucOC), and uncarboxylated prothrombin (PIVKA-II) were analysed. Results: Preoperatively, 29 patients had dp-ucMGP levels above the reference values. Patients with pre-existing cardiovascular comorbidities had higher dp-ucMGP preoperatively compared with patients with no record of cardiovascular disease. Postoperatively, this number increased to 36 patients, and median dp-ucMGP levels increased (*p* < 0.0001) and correlated to a PIVKA-II increase (*r* = 0.44). On the other hand, dp-cMGP levels did not significantly alter. Decreased levels of ucOC and cOC were seen after surgery (*p* = 0.017 and *p* = 0.0033, respectively). Comorbidities, possible nutritional defects, and complications affecting Gla protein activity and function were identified. Conclusions. Dp-ucMGP was high preoperatively, and had further increased postoperatively. This pattern was linked to several comorbidities, possible nutritional defects, and postoperative complications, which motivates further research about potential interactions between perioperative corrective treatments with vitamin K supplements, cardiovascular biomarkers, and incidents of stroke and myocardial infarction events.

## 1. Introduction

Almost a century ago, vitamin K was identified by the Danish researcher Henrik Dam as a key factor in haemostasis [1]. Vitamin K functions as a cofactor in the carboxylation of hepatic coagulation factors II, VII, IX, and X, as well as proteins C, S, and Z, and induces a conformational change that gives these proteins a calcium-dependent affinity for negatively charged phospholipids. The vitamin K dependent proteins are collectively referred to as Gla proteins, relating to their glutamate (Glu) residues that are carboxylated (Gla). 

To date, more than 14 Gla proteins originating from the liver, as well as from other tissues, have been identified [2]. Two well-known extrahepatic proteins are the matrix Gla protein (MGP) and osteocalcin (OC); OC is also known as bone Gla-protein (BGP). Although OC and MGP have a relatively high degree of sequence similarity and share a high affinity for hydroxyapatite (HA) crystals, they have different functions in the body and originate from different cell types [3].

MGP is mainly expressed by fibroblasts, chondrocytes, and vascular smooth muscle cells (VSMC), and functions as an inhibitor of ectopic calcification [4]. The significance of MGP for vascular health was demonstrated by generating MGP knock-out mice, which died from aortic rupture before reaching two months of age [5]. Ectopic secretion of MGP from the liver did not reverse the calcification. However, with local knock-in of MGP, the vascular abnormalities were avoided, indicating that the process is regulated locally and not systemically [6]. In addition to carboxylation, post-translational phosphorylation can modify MGP, and is believed to affect transportation routes and secretion rates to extracellular surroundings [7]. Hence, MGP may be present in the circulation in different states of carboxylation and/or phosphorylation. Desphospho-uncarboxylated MGP (dp-ucMGP) has been associated with increased arterial calcification and stiffness [8]. A suboptimal function with regard to protection against tissue mineralization by MGP (by impaired carboxylation, for example), might increase the risk in postoperative patients of future cardiovascular events, but this is an unexplored area. This would be especially interesting to study in patients with advanced arteriosclerotic manifestations [9], type II diabetes [10], and chronic kidney disease [11] who have increased plasma levels of dp-ucMGP.

OC was initially recognised as a regulator of bone mineralisation, and has recently been associated with extra-skeletal effects, such as an involvement in glucose metabolism and male fertility [12].

We have recently published a study on early perioperative changes in hypocarboxylated prothrombin, as measured by proteins induced by vitamin K absence for factor II (PIVKA-II), which supported the hypothesis that perioperative vitamin K deficiency is common, but not detected by different prothrombine time (PT) methods [13]. Previous research has confirmed a positive association between PIVKA-II and the percentage of uncarboxylated OC (uOC/OC) in a healthy adult population, and this ratio can be used to evaluate vitamin K nutritional status [14]. Increased plasma levels of both dp-ucMGP and PIVKA-II have been reported in patients with end stage renal disease [15].

To date, perioperative changes in extrahepatic vitamin K status are an unexplored field of research. Surgical patients are at risk for malnutrition during the postoperative period, which in combination with the complex metabolic response to surgical stress, may lead to adverse outcomes [16]. Previous studies have shown that plasma vitamin K1 levels are significantly reduced after a few days with low intake, with subsequent development of subclinical hepatic vitamin K deficiency [17]. The primary aims of this observational screening study was to measure the different variants of MGP and OC preoperatively and up to five days postoperatively in patients scheduled for abdominal or orthopaedic surgery to determine whether an extrahepatic vitamin K deficiency develops.

## 2. Materials and Methods

### 2.1. Patients

All patients gave their informed and written consent to participate, and both the study and the Biobank were approved by the Regional Ethical Board, Lund, Sweden (ethical approval code DNR 374). The work was carried out in accordance with the Code of Ethics of the World Medical Association (Helsinki Declaration) of 1975, as revised in 1983. The study was a prospective screening study, and included patients undergoing elective and emergency gastrointestinal, urological, or orthopaedic surgery. No power analysis was performed.

In order to create a homogenous study population with respect to liver function and bleeding tendencies, patients with hepatocellular carcinoma, icteric liver disease, hepatitis, liver cirrhosis, known bleeding disorders, elevated plasma bilirubin or plasma liver transaminases, or treatment with either strong platelet inhibitors or warfarin with a prothrombin time with international normalized ratio (PT-INR) of more than 1.2 were excluded from participation.

### 2.2. Sampling and Storage

Venous blood for the analysis of vitamin K-dependent proteins was sampled into citrated tubes immediately before surgery (day 1) and in the morning four days after surgery (day 5). The samples were instantly centrifuged, as described previously [13].

### 2.3. PIVKA-II

Plasma PIVKA-II concentrations were measured with ELISA using a commercially available kit (Diagnostica Stago, Asnière, France) [18]. The test was sensitive to PIVKA-II concentrations >2 µg/L. The perioperative PIVKA-II changes have already been published together with the activated prothrombin time (aPTT) and 2 different PT methods: Quick-PT and Owren-PT changes from these patients [13]. Thus, only the correlations between PIVKA-II and the extrahepatic Gla proteins fractions are presented here and discussed against individual extrahepatic Gla protein changes.

### 2.4. MGP

MGP sandwich ELISAs for detecting dp-ucMGP and desphospho-carboxylated MGP (dp-cMGP) were developed at VitaK BV, Maastricht, the Netherlands [19]. In brief, circulating dp-ucMGP was quantified in citrated plasma samples using the dual antibody sandwich ELISA. In this assay, the capture antibody is a monoclonal antibody directed against the non-phosphorylated sequence 3–15 in human MGP, whereas the second antibody is directed against the non-carboxylated MGP sequence 35–49. For measuring circulating dp-cMGP a monoclonal antibody directed against the carboxylated MGP sequence 35–53 was used. The intra- and inter-assay variation coefficients for dp-ucMGP were 7.6% and 6.8%, respectively; those for dp-cMGP were 9.4% and 8.5% respectively. In a previous study, the reference range was defined for dp-ucMGP as 447 ± 188 pM (mean ± standard deviation), and for dp-cMGP as 1763 ± 478 pM, for a healthy 25 to 80-year-old population [19]. Another study defines the dp-ucMGP range for healthy volunteers as 50–750 pM; and for haemodialysis patients as 1868 ± 870 pM [20]. Furthermore, dp-ucMGP levels above 437 pM have been reported as a risk factor for cardiovascular calcification and mortality [21]. In this study, the reference ranges were chosen as 447 ± 188 pM and 1763 ± 478 pM for dp-ucMGP and dp-cMGP, respectively.

### 2.5. OC

In serum, osteocalcin species were tested based on the enzyme immunoassays uncarboxylated osteocalcin (ucOC) and carboxylated osteocalcin (cOC), which were both obtained from Takara Shuzo Co. Ltd., Shiga, Japan, and carried out according to the manufacturer’s protocol. The intra- and inter-assay variation coefficients for ucOC were 5.2% and 8.5%, respectively; those for cOC were 6.9% and 9.4%, respectively. The reference range for ucOC has been defined as 2–4 ng/mL, and for cOC as 5–9 ng/mL in healthy subjects aged above 40 years. The reference range for ucOC/cOC has been defined to <1.0.

### 2.6. Comorbidities

Patient comorbidities were retrieved from medical journals and at the consent information talk. Comorbidities were: atherosclerotic diseases (stroke (CVA), myocardial infarction (MI), sclerotic aortic valve disease (AVS), coronary artery bypass grafting (CABG), abdominal aortic aneurysm (AAA), peripheral arterial atherosclerotic disease, and percutaneous coronary invention (PC)); diabetes mellitus (DM) type II; and kidney disease with creatinine clearance <30 mL/min/1.73 m^2^ and infection signs such as high C reactive protein (CRP), sepsis with positive blood culture, peritonitis, or other signs of infection. Other diseases that may decrease carboxylation are included, such as psoriasis, rheumatoid arthritis, and ulcerative colitis. No diet inquiries were performed; therefore, the vitamin K intake prior to surgery is unknown. 

### 2.7. Gastrointestinal Recovery Time—GIRT

Gastrointestinal recovery time (GIRT) is defined as full oral nutrition and the passage of normal stool; this correlated to high PIVKA-II in singular patients from the present patient material, as has been previously published [13]. Full oral nutrition, the passage of normal stool, and the retention of gastrointestional fluid when checking volume intermittently sucked from the nasogastric tube (in patients with such) and passage of normal stools—were registrated in daily nurse charts. 

### 2.8. Statistics

Data processing was performed using GraphPad Prism ((GraphPad Software, La Jolla, CA, USA) for Windows, version 7. Variables were non-parametric, and Wilcoxons signed-rank test was used for comparisons between preoperative (day 1) and four days postoperative (day 5). A *p*-value of < 0.05 was considered statistically significant. Correlations were analysed using Spearman’s correlation test.

## 3. Results

### 3.1. Patient Characteristics

The present study included 40 patients undergoing elective or emergency gastrointestinal, urological, or orthopaedic surgery. The study population consisted of 12 female and 28 male patients with a median age of 71 (range 32–88) years. The patients were divided into subgroups based on the anatomical location of their condition; 23 patients underwent abdominal or urological surgery (A), and the remaining 17 patients underwent orthopaedic surgery (O). Thirty-nine of these patients have previously been described [13]. The present study involves an extra patient no. 41 (right hemicolectomy) to compensate for the loss of one patient due to problems with venous access postoperatively, as described in the previous PIVKA-II study (39 patients). For technical reasons, it was not possible to analyse MGP in one patient, and in another patient, it was not possible to analyse OC.

### 3.2. MGP

**Figure 1:**
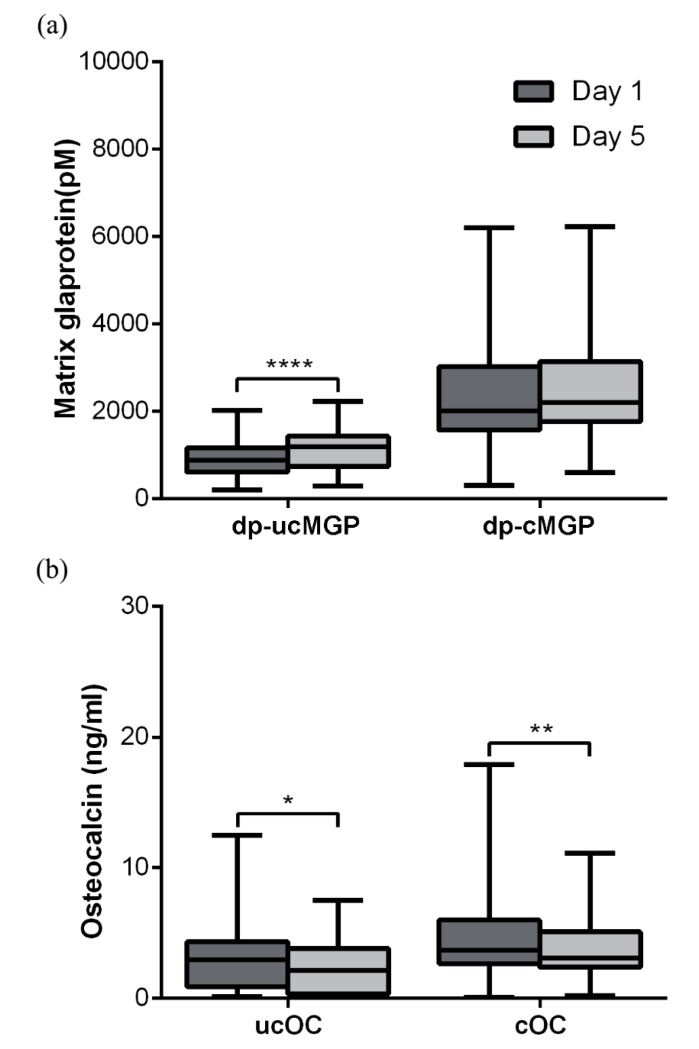
(**a**,**b**) Boxplot changes in matrix Gla protein (MGP) and Osteocalcin (OC), where dp-ucMGP = desphospho-uncarboxylated matrix Gla protein, and dp-cMGP = desphospho-carboxylated matrix Gla protein; and osteocalcin (OC), where ucOC = uncarboxylated osteocalcin, and cOC = carboxylated osteocalcin. Significance levels: * *p* < 0.05, ** *p* ≤ 0.01 and **** *p* ≤ 0.0001.

**Figure 2:**
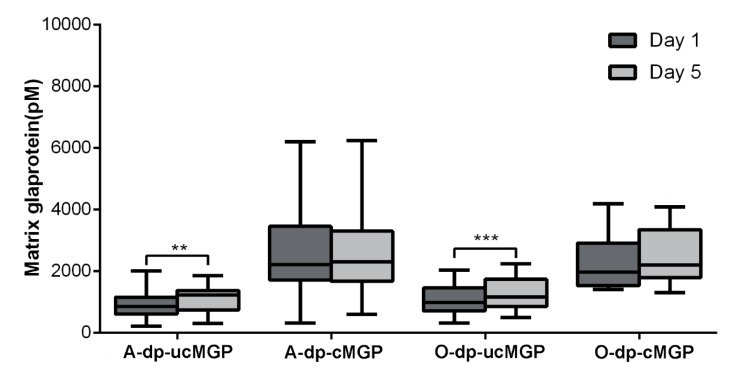
Boxplot changes in MGP before surgery and five days postoperatively (A = abdominal surgery, O = orthopaedic surgery, dp-ucMGP = desphospho-uncarboxylated matrix Gla protein, and dp-cMGP = desphospho-carboxylated matrix Gla protein). Significance levels: ** *p* ≤ 0.01 and *** *p* ≤ 0.001.

Plasma levels of dp-ucMGP had increased significantly at day 5 compared with day 1 for all of the patients combined, with *p* < 0.0001 (Figure 1a), and in the abdominal and orthopaedic subgroups separately, with *p* = 0.0083 and *p* = 0.0007, respectively (Figure 2). Dp-cMGP had not significantly altered when comparing day 5 with day 1 (Figure 1a). Twenty-nine of the 39 patients (74%) had a dp-ucMGP above the reference range at day 1 (median 887 pM, range 210–2027 pM). At day 5, only four patients (10%) had dp-ucMGP within the reference interval assessed in the general population (median 1186 pM, range 301–2233 pM). In patients with pre-existing cardiovascular comorbidities (*n* = 10), dp-ucMGP was more significantly increased both at baseline (median 1377 pM, range 587–2008 pM) and at day 5 (Median 1433 pM, range 300–1848 pM). However, the increase between baseline and day 5 was not statistically significant. Cardiovascular comorbidities included a previous CABG, myocardial infarction, aortic stenosis, angina, atrial fibrillation, and cardiac failure. Eighteen of the 39 patients (46%) had a dp-cMGP above the reference range (>2241 pM) at day 1. At day 5, 19 patients (49%) had dp-cMGP above the reference interval assessed in the general population. Table S1 (abdominal patients) and Table S2 (orthopedic patients) define patients with comorbidities, and dp-ucMGP > 750 pM results are marked with bold face. Only one of the 17 patients in the orthopedic group, and four of the 23 patients in the abdominal group, had a decrease in dp-ucMGP at day 5 as compared with day 1 (results underlined in Tables S1 and S2).

### 3.3. OC

**Figure 3:**
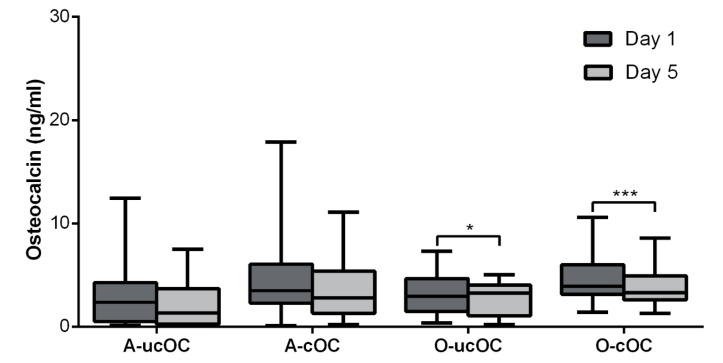
Boxplot changes in OC concentrations over time (A = abdominal surgery, O = orthopaedic surgery, ucOC = uncarboxylated osteocalcin, and cOC = carboxylated osteocalcin). Significance levels: * *p* < 0.05 and *** *p* ≤ 0.001.

Plasma levels of ucOC at day 5 compared with day 1 revealed a decrease for all of the patients with *p* = 0.017 (Figure 1b), and in the orthopedic subgroup (O-ucOC), with *p* = 0.044 (Figure 2). All of the patients in the orthopaedic subgroup had decreased cOC at day 5, with *p* = 0.0033 and *p* = 0.0010, respectively (Figure 1b and Figure 3; Table S2). The ratio of uncarboxylated OC to the carboxylated OC plasma concentrations did not change from day 1 to 5 in all of the patients or in any of the subgroups. Six of the 23 (26%) abdominal patients (one with a very high ucOC of 12.47) had ucOC levels above the upper normal range (>4 ng/mL) before surgery (Table S1), as did six of the 16 (38%) patients in the orthopaedic subgroup (Table S2). After surgery, four patients in the abdominal subgroup (17%) and four patients in the orthopaedic subgroup (25%) had ucOC levels above the upper normal range. Three of the 39 patients had cOC levels above the upper normal range (>9 ng/mL) preoperatively. After surgery, only one patient had cOC levels above the upper normal range. Nine patients (23%) had an ucOC/cOC ratio above the upper reference value (>1.0) preoperatively, and 10 patients (26%) had an ucOC/cOC ratio above the upper reference value postoperatively.

### 3.4. Correlations

No significant correlations were found between any of the MGP or OC parameters. Dp-ucMGP showed no significant correlation with PIVKA-II on day 1. However, at day 5, dp-ucMGP showed a significant correlation with PIVKA-II (Spearman coefficient of 0.44; *p* = 0.005). No such association could be found between any of the OC species and PIVKA-II. The correlation analyses are summarized in Table 1. There were no correlations between intraoperative blood losses and the different Gla proteins, nor when subgroup analyses in A or O patients were performed.

### 3.5. Comorbidities and MGP and OC Changes

Postoperative changes in the plasma analyses of dp-ucMGP or ucOC above the reference ranges as defined for healthy individuals could be correlated to various comorbidities, as defined in the method section, which could potentially decrease the carboxylation degree of the Gla proteins. These changes are presented in Tables S1 and S2.

### 3.6. Postoperative Complications and MGP and OC Changes

Postoperative complications that could potentially decrease the carboxylation degree of Gla proteins are presented in Tables S1 and S2; examples are wound infection, postoperative nausea, paralytic ileus, delayed gastrointestinal recovery time (GIRT), pain > 6 on the visual analogue scale (VAS), and problems with enteral nutrition. GIRT was defined as full oral nutrition and the passage of normal stool, as previously defined and presented in our published PIVKA-II data from the same patients as in the present study [13]. In addition, gastrontestinal retention fluid volumes were also addressed. There were no clinical signs of postoperative stroke or myocardial infarction episodes.

## 4. Discussion

In this study, we monitored postoperative changes in the extrahepatic Gla proteins MGP and OC in surgical patients. Preoperative dp-ucMGP was well above the reference range in most of the patients, and further increased early postoperatively in the majority of the patients. At baseline, median dp-ucMGP was higher than observed in previous studies on healthy individuals [22]. This may in part be explained by an age-dependent increase in dp-ucMGP, as our study population were older, but may also reflect an overall poorer health. In patients with increased cardiovascular risk, dp-ucMGP was more markedly elevated. In these patients, dp-ucMGP was higher (>977 pM) than reported in previous studies [9]; however, the number of patients with pre-existing cardiovascular disease was very limited in the present study. UcOC was elevated in only a few patients preoperatively, and decreased after surgery. Many patients had comorbidities that previously have been shown to be associated with elevated dp-ucMGP, such as earlier myocardial infarction, diabetes type II, and chronic kidney dysfunction. The data in the present study demonstrate that in this subset of surgical patients, vascular vitamin K status is poor.

Deviating levels of MGP species were found in a large fraction of the patients with cardiovascular comorbidities (Tables S1 and S2). Elevated dp-ucMGP has been previously reported in patients with atherosclerosis [9], and might reflect an increased production and release of MGP to counteract the atherogenesis. Furthermore, dp-ucMGP has been associated with myocardial failure assessed by N-terminal prohormone of brain natriuretic peptide (NT-proBNP) and left ventricular ejection fraction, suggesting that MGP might be involved not only in vascular calcification, but also in myocardial remodelling and the development of heart failure [23]. Several preclinical and observational studies have demonstrated a reduced progression and even the reversal of vascular calcifications after vitamin K supplementation, which has motivated several clinical trials [24]. Among these is the VitaK-CAC trial, where patients with pre-existing coronary artery calcification (CAC) are supplemented with vitamin K2 subspecies Menaquinone-7 (MQ-7) for 24 months and evaluated with multi-sliced computed tomography to determine whether supplementation affects the rate of CAC progression [25]. Vitamin K deficiency can be exacerbated by drugs such as warfarin (see method for exclusion criteria) or statins, which appear to interfere with vitamin K recycling and the in vivo synthesis of vitamin K2 [26]. In the present study, nine patients were treated with statins. A recent in vitro study demonstrated that statins inhibit the accumulation of MK-4 (vitamin K2 subspecies), which is otherwise seen in human VSMCs pretreated with vitamin K3 [27]. Authors hypothesised that the statin-induced inhibition of vitamin K2 synthesis contributes to the lack of survival benefit of statins in patients with end stage renal disease. 

Seventeen patients had different types of cancer. The prolonged suboptimal function of Gla proteins can increase the risk of cancer, as suggested by studies where vitamin K intake was inversely correlated to cancer incidence [28,29], as well as by the documented anti-carcinogenic effects of vitamin K [30]. The expression of MGP has been investigated in several cancer types, but no clear role has been established [31]. Several studies indicate that the daily intake of vitamin K is not sufficient to ensure optimal carboxylation status among the hepatic or extrahepatic vitamin K-dependent proteins [32,33]. This, as well as the already described comorbidities, might explain why many of our patients had elevated levels of dp-ucMGP, ucOC, and PIVKA-II before surgery. It is also possible that the results would be different if sampling had been conducted over an extended postoperative period since the extrahepatic tissues mostly utilize vitamin K2, which has a longer half-life. It seems that the levels of vitamin K do not always correlate in the hepatic and extrahepatic tissues. Osteocalcin, which is a specific marker for bone metabolism, is not affected as much as PIVKA-II (liver) and dp-ucMGP (vascular wall). It is possible that perioperative bedrest decreases bone metabolism, which causes OC levels (both ucOC and cOC) to decline. Nevertheless, since dp-ucMGP is increased postoperatively, further studies determining whether this poses an increased risk for postoperative stroke or myocardial infarction are needed. In a previous study, vitamin K deficiency was induced in healthy subjects by feeding them a diet low in vitamin K1 for two weeks [17]. It only took a few days to reduce plasma vitamin K1 levels by half, and by the end of the depletion period, plasma PIVKA-II levels had significantly increased, suggesting hepatic vitamin K deficiency. These results suggest that a subclinical vitamin K deficiency may develop over the course of a few days in subjects with low intake. In our study, we demonstrate that similarly, dp-ucMGP increases in the postoperative setting, indicating that vascular vitamin K deficiency may also develop or worsen quickly. Whether prolonged elevation of dp-ucMGP poses an increased cardiovascular risk is unclear. Some studies report that high levels of dp-ucMGP are independently associated with an increased risk for cardiovascular disease [34], whereas others do not support this finding [35]. Future studies identifying racial, ethnical, age, and gender disparities are needed to better clarify the diagnostic utility of dp-ucMGP.

This study has several limitations. The sample size is small and heterogenous. Also, a control group with minor surgery, such as breast surgery, would be interesting for comparison. Longer follow up with sampling after four weeks would indicate in what direction the MGP and OC fractions and PIVKA-II had changed. Measuring cardiovascular biomarkers such as troponin, NT-proBNP, neuron-specific enolase (NSE), and the registration of stroke and myocardial infarction events would be interesting. We did not have any clinical events in our patients, but cannot rule out subclinical ischemic episodes. The analyses performed in this study can only be performed in highly specialized laboratories and are costly. A more discriminative approach would be to study patients with ischemic events and match them against patients without, and then measure MGP changes in both groups. In accordance with previous research, this study supports that dp-ucMGP is elevated in patients with cardiovascular disease. Furthermore, this study is the first to demonstrate postoperative changes in dp-ucMGP. Taken together, an improved study design would comprise a larger number of age-matched participants with similar cardiovascular risk. 

## 5. Conclusions

In summary, our results demonstrate a preoperative elevation in dp-ucMGP that further increases postoperatively. This pattern is linked to several comorbidities and postoperative complications, and motivates further research about interactions between perioperative corrective treatment with different types of vitamin K supplements, cardiovascular risk factors and events, and biomarkers of vascular ischemia or heart failure.

## Figures and Tables

**Table 1:** Spearman correlations between different Gla-protein (Gla-prot) fractions (d1 = day 1, d5 = day 5, dp-ucMGP = desphospho-uncarboxylated matrix Gla protein, dp-cMGP = desphospho-carboxylated matrix Gla protein, ucOC = uncarboxylated osteocalcin, and cOC = carboxylated osteocalcin).

Gla-prot	Dp-ucMGP.d1	Dp-ucMGP.d5	Dp-cMGP.d1	Dp-cMGP.d5	ucOC.d1	ucOC.d5	cOC.d1	cOC.d5	PIVKA-II.d1	PIVKA-II.d5
Dp-ucMGP.d1	1.00	0.79	0.50	0.49	0.07	−0.04	−0.18	−0.13	0.31	0.38
Dp-ucMGP.d5	0.79	1.00	0.29	0.48	−0.11	0.04	−0.19	−0.06	0.15	0.44
Dp-cMGP.d1	0.50	0.29	1.00	0.86	0.07	−0.17	0.05	−0.04	0.16	−0.02
Dp-cMGP.d5	0.49	0.48	0.86	1.00	−0.04	−0.06	0.14	0.17	0.10	−0.07
ucOC.d1	0.07	−0.11	0.07	−0.04	1.00	0.68	0.55	0.47	−0.02	−0.09
ucOC.d5	−0.04	0.04	−0.17	−0.06	0.68	1.00	0.36	0.44	−0.06	−0.10
cOC.d1	−0.18	−0.19	0.05	0.14	0.55	0.36	1.00	0.84	−0.11	−0.12
cOC.d5	−0.13	−0.06	−0.04	0.17	0.47	0.44	0.84	1.00	−0.24	−0.20
PIVKA-II.d1	0.31	0.15	0.16	0.10	−0.02	−0.06	−0.11	−0.24	1.00	0.52
PIVKA-II.d5	0.38	0.44	−0.02	−0.07	−0.09	−0.10	−0.12	−0.20	0.52	1.00

## Supplementary Materials

The following are available online at www.mdpi.com/2072-6643/10/1/46/s1, Table S1: Characteristics of patients undergoing abdominal surgery. (D1 = day 1, D5 = day 5, MI = myocardial infarct, PCI = percutaneus coronary intervention, HT = hypertension, DM = diabetes mellitus, CABG = coronary artery bypass grafting, CVA = cerebral vascular accident, AVS = aortic valve stenosis, PE = pulmonary embolism, GIRT = gastrointestinal recovery time, CKD = chronic kidney disease, COPD = chronic obstructive pulmonary disease, CHF = congestive heart failure, Afib = atrial fibrillation and AAA = abdominal aortic aneurysm; Table S2: Characteristics of patients undergoing ortopaedic surgery. (D1 = day 1, D5 = day 5, CVA = cerebral vascular accident, DM = diabetes mellitus, HT = hypertension, PPI = proton pump inhibitor, MI = myocardial infarct, Afib = atrial fibrillation, GI = gastrointestinal, GIRT = gastrointestinal recovery time, CHF = congestive heart failure and AVS = aortic valve stenosis).
