# Supplementary material for: Desphospho-Uncarboxylated Matrix-Gla Protein Is Increased Postoperatively in Cardiovascular Risk Patients
Source: Nutrients. 2018 Jan 5;10(1):46. doi: 10.3390/nu10010046 (PMC5793274; doi:10.3390/nu10010046)
Supplement: Supplementary file 1 [file nutrients-10-00046-s001.docx]

Table S1.

| **Patient Male/Fem** | **Age** | **Surgery** | **Dp-ucMGP**  **D1/D5**  **PIVKA-II>30μg /L** | **ucOC**  **D1/D5** | **Comorbidity** | **Postop compl** |
| --- | --- | --- | --- | --- | --- | --- |
| 1(M) | 32 | Ileal pouch-anal anastomosis | 401**/594** | **5,89/7,52** | Ulcerative colitis | None |
| 2(M) | 71 | Abdomino-perineal resection | **1004/997** | **4,31**/1,86 | Faecal incontinence | None |
| 3(M) | 71 | Sigmoid resection | **914/1434** | 1,48/**4,22** | MI (PCI), HT, asthma,  gout, hyperrlipidemia | Wound infection,  antibiotics |
| 4(M) | 72 | Right hemicolectoy | **887/1039** | 0,46/0,57 | HT, kidney stones | None |
| 5(F) | 62 | Explorative laparatomy on suspicion of diverticulitis- negative no resection | 210/701 | 1,58/0,64 | Asthma, IgG defect, osteopenia, bowel dysfunction | Nausea |
| 7(M) | 69 | Emergency open cholecystectomy  CT okey, CRP>200 mg/L, no fever | **912/749** | **4,07**/0,27 | Unexpected peritonitis due to perforated gallbladder | Expedient recovery  broad spectrum antibiotics |
| 8(M) | 62 | Prostatectomy | 329/437 | 1,00/1,35 | HT, DM type II,  hyperlipidemia | None |
| 9(M) | 79 | Explorative laparotomy:  Sigmoid resection and part of jejunum due to inflammatory mass | 607**/1186**  **PIVKA D5** | 0,77/2,78 | CVA, AVS,  hyperlipidemia, PE,  preop broad spectrum | Delayed GIRT |
| 10(M) | 81 | Anterior resection | 604**/868** | 2,60/0,23 | Peripheral arteriosclerosis,  angina pectoris,  macula degeneration bilat., hyperlipidemia | Delayed GIRT |
| 12(M) | 71 | Prostatectomy | 611**/**746 | 3,87/3,68 | HT | Wound infection, delayed GIRT, |
| 13(M) | 82 | Right hemicolectomy + distal jejunum | **2008/1425**  **PIVKA DI/D5** | 0,12/0,08 | Several MIs (CABG),  preop sepsis with clostridium septicum | Paralytic ileus, delayed GIRT  postop sepsis with clostridium septicum |
| 14(M) | 71 | Whipple´s procedure | **-/-** | 0.29/0,25 | MI (PCI) | None |
| 16(M) | 81 | Nephroureterectomy | **1227/1321** | **4,75**/2,31 | Kidney dysfunction,  preop urosepsis | Nausea, delayed GIRT |
| 17(M) | 74 | Prostatectomy | **1377/1753** | 1,18/0,44 | MI (CABG),  kidney dysfunction, DM | None |
| 19(M) | 68 | Left hemicolectomy +  Distal pancreasresection | **1171/1271**  **PIVKA D1/D5** | **12,47/4,89** | Hyperlipidemia,  atrial fibrillation, warfarin stopped preop | Delayed GIRT |
| 20(F) | 71 | Right hemicolectomy | 732**/1319** | 3,25/2,53 | CKD | Nausea |
| 26(M) | 62 | Right hemicolectomy | **860/704** | 0,42/0,32 | None | None |
| 30(M) | 85 | Sigmoid resection | **1146/1703** | 3,63/3,18 | Prostatic cancer – antiandrogenic therapy | None |
| 34(M) | 69 | Cystectomy +Bricker | **836/1320** | 2,16/3,81 | HT | None |
| 37(M) | 72 | Explorative laparotomy  Peritonitis, CRP>350 mg/L | **1459/1848**  **PIVKA D5** | 0,18/0,20 | COPD, CHF, Afib,  arterial embolism, pain,  malnutrition, hyperlipidemia | Pain, paralytic ileus, delayed GIRT,  faecal C.difficile  wound infection |
| 38(M) | 68 | Gastric resection | 588/301 | 3,92/0,21 | AAA resection, CABG | None |
| 39(M) | 88 | Anterior resection | 641**/1321** | **4,39/4,88** | None | None |
| 41(F) | 87 | Right hemicolectomy | **852/1354** | 0,25/0,23 | RA, osteoporosis | Nausea |

Table S1- Characteristics of patients undergoing abdominal surgery. (D1 = day 1, D5 = day 5, MI = myocardial infarct, PCI = percutaneus coronary intervention, HT = hypertension, DM = diabetes mellitus, CABG = coronary artery bypass grafting, CVA = cerebral vascular accident, AVS = aortic valve stenosis, PE = pulmonary embolism, GIRT = gastrointestinal recovery time, CKD = chronic kidney disease, COPD = chronic obstructive pulmonary disease, CHF = congestive heart failure, Afib = atrial fibrillation and AAA = abdominal aortic aneurysm.

Table S2.

| **Patient Male/Fem** | **Age** | **Surgery** | **Dp-ucMGP**  **D1/D5**  **PIVKA-II>30μg/L** | **ucOC**  **D1/D5** | **Comorbidity** | **Postop compl** |
| --- | --- | --- | --- | --- | --- | --- |
| 6 (M) | 68 | Hip-prothesis | **913/1161** | 3,67/**4,07** | CVA, hyperlipedemia | None |
| 11(F) | 76 | Hip-prothesis | **780/896**  **PIVKA D1** | 2,96/3,62 | Untreated DM type II, pain | Pain |
| 15(M) | 60 | Hip-prothesis | **785/943** | **4,47/4,21** | Psoriasis, hypothyreos | None |
| 18(M) | 64 | Hip-prothesis | **1948/801** | **7,00**/3,56 | Angina pectoris | None |
| 21(F) | 63 | Hip-prothesis | 582/680 | 2,28/2,13 | HT, hyperlipidemia | None |
| 22(F) | 87 | Hip-fracture | **815/1723** | 0,44/0,35 | DM type II | Confusion |
| 23(M) | 74 | Hip-prothesis | **1008/1160** | 3,16/2,08 | Longstanding severe pain from hip | None |
| 25(F) | 78 | Hip-fracture | **2027/2233**  **PIVKA D5** | 2,45/1,00 | RA/Sjogrens syndrome, osteoporosis, HT, using PPI and  high dose paracetamol | None |
| 27(M) | 83 | Hip-fracture | **1556/1828** | 1,16/- | MI | None |
| 28(F) | 88 | Hip-fracture | **1545/2067**  **PIVKA D5** | **7,30**/3,46 | Afib, HT | Confusion,  GI bleeding, paralytic ileus, delayed GIRT |
| 29(F) | 64 | Hip-prothesis | 309/741 | 2,96/**4,66** | HT, psoriasis | None |
| 31(M) | 80 | Hip-prothesis | 637/**920** | 1,79/1,31 | None | None |
| 32(M) | 75 | Hip-prothesis | **993/1203** | **4,84**/3,10 | CVA, HT, gastritis, hyperlipidemia | None |
| 33(F) | 64 | Hip-prothesis | 470/498 | 0,56/0,22 | Asthma, gastritis | None |
| 35(M) | 61 | Hip-prothesis | **985/1303** | **4,27**/3,99 | CHF, HT | None |
| 36(F) | 77 | Hip-prothesis | **1513/1743** | **6,84/5,05** | MI, osteoporosis | None |
| 40(F) | 87 | Hip-prothesis | **1392/1484** | 0,36/0,39 | Mb Bechterew, AVS (mild), osteoporosis | None |

Table S2 - Characteristics of patients undergoing ortopaedic surgery. (D1 = day 1, D5 = day 5, CVA = cerebral vascular accident, DM = diabetes mellitus, HT = hypertension, PPI = proton pump inhibitor, MI = myocardial infarct, Afib = atrial fibrillation, GI = gastrointestinal, GIRT = gastrointestinal recovery time, CHF = congestive heart failure and AVS = aortic valve stenosis).
